# Proceedings of the Charaka Club

**Published:** 1903-02

**Authors:** 


A Monthly Review of Medicine and Surgery.

*EDITOR:*

WILLIAM WARREN POTTER, M. D.

All communications, whether of a literary or business nature, books for review and exchanges should be addressed to the editor: 284 FRANKLIN STREET, BUFFALO, N. Y.

VOL. XLII.—LVIII. FEBRUARY, 1903. No. 7.

### Proceedings of the Charaka Club.[1]

THE Charaka Club, of New York, has after an existence of four years published a volume of its transactions and we were among the fortunate few to receive a copy. Fortunate because of this book the Charaka Club has printed and published for its members and friends only 300 copies.

The club is small and select. Its members are Drs. Pearce Bailey, John S. Billings, John Winters Brannan, Joseph Collins, Charles L. Dana, Arpad G. Gerster, Ward A. Holden, Frederick Peterson, B. Sachs and George F. Shrady. The only honorary member is Dr. William Osler. The object of the club is to cultivate the literary, artistic, and historical aspects of medicine. How well they have succeeded, alas! only a very few are privileged to know. The articles selected for publication are only eight.

The first article on Hindoo medicine is full of interest and instruction in the practice as taught by Charaka. According to Sachs he became the instructor of practitioners upon earth and was the author of the oldest work on the subject of Hindoo medicine.

There are many gems in this paper gathered from the sage. We must content ourselves with a small selection.

A good teacher is like rain falling upon the germinating seed and should possess the following qualifications. He should be kind and humble to everyone. He should always be increasing his knowledge of books, and should be neither angered by the improprieties of others, nor fatigued by their importunities. He should be kind and considerate to his pupils and be able to explain the most complicated statements in the simplest and most perspicuous language. Among the Hindoos, there is a

---

1. Volume I. William Wood & Co.

great veneration for the principles of medicine as handed down from generation to generation.

The article, which is a long one, abounds in quotations showing the practice as applied to fevers, consumption, and directions for operation.

The contributions by Dr. Peterson are three short poems—Heredity, Environment, and Solitude. We will not attempt a description, but with his permission we hope to publish them in some future number of the JOURNAL, for we need not only more science but more—much more—sweetness and light in our lives.

Dr. Gerster contributes an entertaining review of the Hippocratic doctrine of the injuries of the cranium.

Doctor Dana's article on the Cult of Esculapius, his statues and his temple, includes a description and photographs of the antique statues by the Greeks and Romans of Esculapius. The picture of the sacred grove containing the temple, The Tholos, The Abator and the Temple of Artemis, at Epidaurus, is a revelation to the uninitiated of the extent of this ancient and most celebrated of all the structures in which the worship of Esculapius was carried on. It remained a place of worship longer than any other devoted to the god of medicine. To it the Romans sent for the sacred serpent when they, in 270 B. C., adopted Esculapius as one of their gods. It had the finest and largest statue of the god, a great record of cures and a theatre of unrivaled splendor.

From all over the world patients were sent for cure and as its therapeutical resource was almost entirely of the psychical kind, it is perhaps the best illustration of how long a system of suggestive therapeutics, backed by divine authority, can maintain itself against the scepticism and incurable ills of the world.

Dr. Collins contributes two articles, one on hypnotism, not as practised by him but as he is requested to practise it, and a second article on ethics.

The evil spoken of physicians is a charming literary excursion by Dr. Dana in classic fields, and is rich in merry jests of the ancients at our expense. Petrarch said, "The doctors say that life is short, but they appear able to abridge it." Nicocles, 400 B.C., said, "The physician is fortuntate, for the sun exploits his successes and he conceals his errors."

"The maid Calypso often cried
Because she was immortal.
The goose! She surely never tried
The doctor and his bottle."

Addison furnished this on consultations:

A single doctor like a sculler plies,
The patient lingers and by inches dies;
But two physicians, like a pair of oars,
Waft him with swiftness to the Stygian shores.

La Bruyère is kinder when he says: "As long as a man is sick and wants to live, he will employ physicians and abuse them."

What a delightful time the Charakans had at their meetings, and how wise it is for physicians to wander into other fields than the purely practical, is the conclusion reached by reading this book.

---

Dr. W. D. Greene, Commissioner of Health of Buffalo, in his annual report for 1902, calls attention to the necessity of establishing a filtering plant.

He says that while the city water supply can be considered fairly good, it is not wholly free from danger because of the fact that the lake and river are polluted to some extent by the cities that drain into the lake above Buffalo. In part the report says:

So long as the refuse of upper lake cities is emptied into the lake, carried along, and deposited as a sediment, and reagitated and dispersed by continuous storms, the water carried to our intake will exert its influence upon the public health.

The department has no hesitation in stating that the possibility of typhoid fever from lake water must necessarily be present during certain periods of every year; that its cause is one beyond present control, and, therefore, it is pertinent to consider what are the practical means of overcoming the conditions and their results as stated.

The solution of the question will be found in the establishment of filter beds constructed on sound hygienic principles and located after careful engineering inquiry, and so long as the condition exists, it becomes the duty of the department to call attention to it with the suggestion as to its correction.

---

Dr. Adolf Lorenz, who sailed for England, December 31, 1902, has reached his home at Vienna in safety, and has given his countrymen interesting accounts of his American visit. He likes this country and its people and does not hesitate to say so. He is enthusiastic over our physicians and in regard to the great charities that we have established.

Prior to his departure he was officially received at the City Hall, at New York, by the Mayor and corporation, when he was presented with engrossed resolutions and the freedom of the city. At a farewell luncheon served at the Hardware Club, tendered by Aldermen Walkley, Sullivan and Stewart, the committee on the resolutions, a well expressed address was delivered by Mr. Walkley.

Dr. Lorenz replied in a well chosen speech which deserves to be read. He said:

When I first put my foot on this soil three months ago, I had a sense of oppression because of the greatness of this place. A feeling of clumsiness came upon me. I had no idea then of the magnificent leave-taking you have prepared for me in this hour. Nor could I have any idea of the reception that would meet me everywhere in this country, from the Atlantic to the Pacific coast. In olden times the freedom of cities used to be given to princes and victorious warriors. Today you are conferring this freedom of the city upon a poor and humble physician. This makes me think of the great changes that have taken place in the last few centuries. Not only princes are honored today, but every man whose work tends to benefit mankind is, in your eyes, worthy of this honor. I take it gratefully from your hands. Need I assure you that this document will be the most precious reward of my efforts?

This token of your esteem is a proof that in America wealth and position are not esteemed higher than work done for the relief of suffering humanity. This token of your esteem is a further proof that this city is not only unique in its wealth, but also unique in its charities. I rejoice in this great honor all the more because I am far from regarding it as a personal one; but I am proud to belong to a profession to which this honor is due. In honoring me you have honored the profession. I thank you from the bottom of my heart, and I assure you that I leave your glorious country with great regret, and with the highest admiration for America and American people.

Dr. Lorenz's remarks were loudly cheered, and for about ten minutes before leaving the City Hall a large crowd surrounded him, shook him by the hand and bade him God-speed.

